# Severely Disturbed Sleep in Patients With Acute Ischemic Stroke on Stroke Units: A Pilot Study

**DOI:** 10.3389/fneur.2019.01109

**Published:** 2019-10-25

**Authors:** Jeannette Hofmeijer, Ruud van Kaam, Sarah E. Vermeer, Michel J. A. M. van Putten

**Affiliations:** ^1^Department of Clinical Neurophysiology, University of Twente, Enschede, Netherlands; ^2^Department of Neurology, Rijnstate Hospital, Arnhem, Netherlands; ^3^Department of Clinical Neurophysiology, Medisch Spectrum Twente, Enschede, Netherlands

**Keywords:** ischemic stroke, continuous EEG, stroke unit, secondary deterioration, sleep

## Abstract

**Introduction:** Previous studies revealed a high prevalence of sleep-wake disturbances in subacute and chronic stroke. We analyzed sleep quantity and quality in patients with hyperacute ischemic stroke on stroke units.

**Methods:** We categorized sleep stages as N1, N2, N3, and REM according to the 2017 criteria of the American Academy of Sleep Medicine in 23 continuous, overnight EEG registrations from 18 patients, starting within 48 h since the onset of cortical ischemic stroke. Associations between presence and duration of sleep stages, and secondary deterioration or functional outcome were analyzed.

**Results:** Physiological sleep cycles were seen in none of the patients. Otherwise, sleep stages alternated chaotically, both during day- and during nighttime, with a sleep efficiency of 30% and 10.5 ± 4.4 (mean ± SD) awakenings per hour of sleep. We cannot differentiate between stroke related and external factors. Only few interruptions could be related to planned nightly wake up calls, but turbulence on stroke units may have played a role. Six patients (seven nights) did not reach deep sleep (N3), 10 patients (13 nights) did not reach REM sleep. If reached, the mean durations of deep and REM sleep were short, with 37 (standard deviation (SD) 25) and 18 (SD15) minutes, respectively. Patients with secondary deterioration more often lacked deep sleep (N3) than patients without secondary deterioration [4 (57%) vs. 2 (25%)], but without statistical significance (*p* = 0.12).

**Conclusion:** We show that sleep is severely disturbed in patients with acute ischemic stroke admitted to stroke units. Larger studies are needed to clarify associations between deprivation of deep sleep and secondary deterioration.

## Introduction

Sleep-wake disturbances are highly prevalent among stroke survivors, with insomnia being reported in up to half of all patients during the first months (1). Insomnia may be a direct consequence of the infarct or associated with environmental factors (1).

In patients with brain infarction or hemorrhage, insomnia was associated with more severe stroke, less functional recovery, and depression (1). Sleep disordered breathing gave an elevated risk of death or recurrent vascular events (2) and stroke recovery was worse in patients with restless legs syndrome (3). Sleep deprivation augmented brain injury in experimental stroke models (4), and drugs to promote non-rapid eye movement (REM) and REM-sleep had a favorable effect on neuroplasticity (5). This suggests that poor sleep may be a modifiable factor, where sleep enhancement may improve recovery of patients with ischemic or hemorrhagic stroke.

Previous studies have focused on the subacute and chronic phases. We analyzed sleep quantity and quality in patients with hyperacute ischemic stroke on stroke units, and related sleep to measures of functional recovery.

## Methods

### Design

We performed a prospective cohort study with continuous full band electro-encephalography (EEG) in patients with acute cortical ischemic stroke at stroke units of Rijnstate Hospital, Arnhem and Medisch Spectrum Twente, Enschede (February 2016-October 2016). The primary aim of this study was detection of cortical spreading depression (6). Patients were included and followed prospectively, whereas analyses of sleep were done retrospectively.

### Approval and Consent

The Medical Research Ethics Committee Twente approved the research protocol for monitoring and follow up (registry number NL50284.044.14). Informed consent was obtained from the patient or a legal representative.

### Patients

Inclusion criteria were age ≥18 years, clinical symptoms consistent with a cortical localization, and a score of ≥4 on the National Institutes of Health Stroke Scale (NIHSS). Exclusion criteria consisted of any progressive brain illness, expectation of short term death due to stroke, and a pre-stroke modified Rankin Scale (mRS) score of >2. Treatment was according to standard protocols, including early mobilization, if possible. Interference between the EEG and mobilization was minimized by the use of small, mobile EEG equipment.

### Outcome

The primary outcome measure was functional outcome as expressed by the score on the mRS at 3 months. The mRS is a 7-point scale ranging from 0 (no symptoms) to 6 (death). A score of 2 or less indicates functional independence (7). Secondary outcome measures included secondary deterioration during hospital admission, defined as an increase on the NIHSS of two points or more. The NIHSS score was collected at first presentation, at the start of EEG, every morning, at the end of EEG, and with signs of deterioration.

### EEG Registration

Full band EEG started as soon as possible with 48 h after symptom onset, aiming at a minimum of one overnight registration. A Neurocenter EEG system (Clinical Science Systems, Leiden, the Netherlands) or (for mobile patients) a portable Mobita EEG system (TMS-international, Oldenzaal, the Netherlands) was used, with a DC-coupled amplifier and a sample frequency of 256 or 250 Hz. Twenty-one silver/silver chloride electrodes were placed according to the international 10–20 system. Electrodes were attached by collodion glue and filled with 10–20 conductive paste. Electrode impedance was kept below 5 kΩ.

### Analysis

Sleep stages were scored as Non-REM (N)1, N2, N3, and REM sleep by visual inspection of the complete raw EEG data in 30 s epochs according to the 2017 criteria of the American Academy of Sleep Medicine (www.aasmnet.org/scoringmanual/) (8). N1 and N2 were considered “light sleep,” N3 “deep sleep.” Data are presented in a descriptive way. Associations between presence or duration of sleep stages and outcome measures were analyzed with Chi-square, Fisher exact, or Student's *t*-test, where appropriate.

## Results

We collected 23 overnight EEG registrations from 18 patients. Data from one patient (one night) could not be analyzed because of broken electrodes. Baseline characteristics and outcomes are summarized in Table 1. Registration included 252 h of daytime (7 a.m.−10 p.m.) and 193 h of nighttime (10 p.m.−7 a.m.).

**Table 1:** Baseline characteristics and outcome per patient.

**Age (y)**	**Stroke location**	**EEG start (h after stroke)**	**Total EEG monitoring time (h)**	**NIHSS score admission**	**Deterioration time (h after stroke)**	**mRS 3 months**
75–79	MCA-R	1	22	11		4
70–74	MCA-R	18	45	17		5
85–89	MCA-L	17	42	18	22	5
50–54	MCA-R	9	19	10	13	2
70–74	MCA-R	6	64	16		3
70–74	MCA-L	8	21	15	31	3
65–69	MCA-L	5	17	8		2
65–69	MCA-R	18	16	4	11	5
45–49	MCA-L	15	Excluded	21		2
70–74	MCA-R	13	22	11		5
75–79	MCA-L	18	20	12		4
80–84	MCA-L	8	11	10		4
75–79	MCA-R	6	20	15	6	6
85–89	MCA-R	10	21	12		1
70–74	MCA-L	7	26	20	26	6
85–89	MCA-R	35	22	9		6
65–69	MCA-L	7	44	10	23	5
50–54	MCA-L	6	16	23		3

*M, male; F, female; MCA, middle cerebral artery; R, right; L, left; EEG, electroencephalography; NIHS, national institutes of health stroke scale; mRS, modified rankin scale; excluded, excluded from analysis (broken electrodes)*.

Physiological sleep cycles, with REM sleep being followed by N1, N2, and N3 and back, were seen in none of the patients. Otherwise, sleep stages alternated chaotically, both during day- and during nighttime, with a mean of 88 transitions between sleep stages per patient per 24 h, sleep efficiency of 30%, and 10.5 ± 4.4 (mean ± SD) awakenings per hour of sleep. Only few interruptions could be related to nightly wake up calls in the context of stroke care: two in two patients, one in seven patients, and none in the remaining patients.

**Figure 1:**
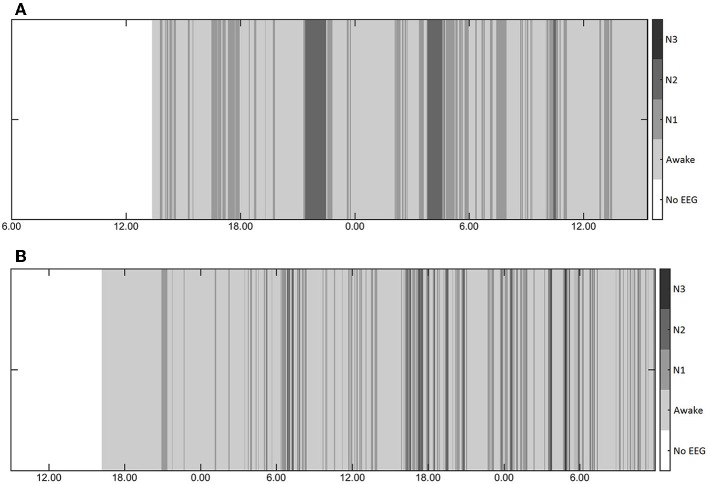
**(A,B)** Typical examples of the course of sleep during two nights in two patients showing lack of physiological sleep cycles, random alterations between sleep stages, and shortness of deep sleep.

Six patients (seven nights) did not reach deep sleep (N3) at all, 10 patients (13 nights) did not reach REM sleep. Typical examples of overnight sleep courses for two patients are shown in Figure 1. Mean duration of each sleep stage (if achieved) per day and night are given in Table 2.

**Table 2:** Sleep efficiency and quantity of sleep per sleep stage.

	**Number of patients that achieved SS**	**Time in SS (if achieved)**	**Total (%)**	**During day (%)**	**During night (%)**
Sleep efficiency			30	30	30
N1	17 (100%)	148 (143)	32	38	62
N2	17 (100%)	260 (206)	55	25	75
N3	11 (65%)	32 (42)	7	8	92
REM	7 (41%)	29 (79)	6	41	59

*Time is given in mean number of minutes (standard deviation). SS, sleep stage; day, 7AM-10PM; night, 10PM-7 AM; Sleep efficiency, total time spent asleep / total tome spent in bed; N1, Non-REM 1 (“light sleep”); N2, Non-REM 2 (“light sleep”); N3, Non-REM 3 (“deep sleep”); REM, Rapid Eye Movement sleep*.

Patients with secondary deterioration more often lacked deep sleep (N3) than patients without secondary deterioration [4(57%) vs. 2(25%)], but without statistical significance (*p* = 0.12). There were no statistically significant associations between other outcome measures and presence or duration of sleep stages.

## Discussion

In this small sample of patients with acute cortical ischemic stroke admitted to stroke units, we show that sleep is severely disturbed. This applied to both quantity and quality of sleep. Approximately half of patients in our study did not reach REM or deep sleep, and if reached, this was mostly of very short duration. Physiological sleep cycles were absent in all participants.

Other than previous studies, we measured sleep in the hyperacute phase and show that in patients with considerable cortical infarcts on stroke units, normal sleep is practically absent. Although sleep time may vary substantially in healthy adults, a total sleep time of approximately 6 h, with 50–53% light sleep, 8–13% deep sleep, 17–20% REM sleep, and 79–84% time asleep while in bed is considered physiological for individuals of 60–80 years old. The observed 27% light sleep, 3% deep sleep, and 2% REM sleep in our study deviate significantly from these physiological values (9). Other publications on the prevalence of sleep-wake disorders after stroke focused on the subacute and chronic phases (1) and revealed insomnia related to central or obstructive sleep apnea in 38–72% of patients (1, 10), restless legs syndrome in up to 13%, mostly combined with periodic limb movements during sleep (3, 11), and REM sleep behavioral disorder in 11% (12). In our relatively small sample, we observed abnormal sleep in all patients.

Sleep-wake disorders, including insomnia, may be a direct consequence of an infarct (13). Otherwise, insomnia can be caused by environmental factors. We cannot differentiate between stroke related and external factors. A relation with nightly turbulence on the stroke unit cannot be excluded. Since disturbed sleep has been consistently associated with elevated risks of death, poor functional recovery and recurrent vascular events in patients with sleep disordered breathing (2), functional recovery was poorer is patients with than without restless legs syndrome (3), and improvement of aphasia has been associated with EEG measures of sleep in patients with insomnia (14), improvement of sleep may be an important modifiable factor to improve recovery of patients with acute brain infarcts.

Sleep enhancement can probably be achieved with relatively simple measures on acute stroke units. Improvement of sleep hygiene, with protection from noise and light, may result in substantial sleep improvement. Sleep disordered breathing can be diagnosed with respiratory polygraphy or nasal airflow measurement and treated with positioning of the patients or continuous positive airway pressure (CPAP). Positional therapy improved apnoe / hypopnoe indices in patients with subacute stroke (15) and transnasal insufflation improved slow wave sleep (16). In the subacute and chronic phases, CPAP treatment was associated with improved neurological recovery (17, 18), less depression (17, 19), and less recurrent vascular events (20). Effects of medication to enhance sleep have shown divergent results, with a tendency toward benefit of sedative antidepressants, zolpidem (1), and dopamine agonists (11), and toward detrimental effects of benzodiazapines (21). Intervention studies in hyperacute stroke are lacking. On a fundamental level, effects of sleep on recovery after brain infarction remain enigmatic. However, accumulating evidence suggests that healthy sleeps enhances neuroplasticity and recovery after ischemic or hemorrhagic stroke (22). Cerebral ischemia leads to subsequent reversible and irreversible synaptic and membrane failure (23). Since beneficial effects of sleep probably include synaptic recovery during REM- and restoration of membrane functioning during deep, non-REM sleep (24), sleep may modulate neuronal stabilization or recovery after an infarct.

Stroke induced EEG changes may hamper correct classification of sleep stages. These include a reduction of sleep spindles and an increase of slow wave activity in both hemispheres (25, 26). Still, sleep analysis after brain infarction or hemorrhage is common, and disturbances of sleep architecture are generally considered an expression of disturbed sleep rather than a direct expression of stroke (22).

Limitations of our study include the small sample size, lack of complete polysomnography's, limited information on lesion location, and lack of a control group. Also, most patients did not have a full 24 h recording. Thereby, we cannot exclude that sleep happened outside the recording hours, but still within 24 h.

Our pilot study suggests that sleep could be disturbed in all patients with acute ischemic stroke. Larger studies are needed to clarify associations between deprivation of deep sleep and secondary deterioration. If associations are confirmed, intervention studies testing effects of sleep enhancing therapies to improve functional recovery would be required.

## Data Availability Statement

The datasets generated for this study are available on request to the corresponding author.

## Ethics Statement

The Medical Research Ethics Committee Twente approved the research protocol for monitoring and follow up (registry number NL50284.044.14). Informed and written consent was obtained from the patient or a legal representative.

## Author Contributions

JH: study design, conceptualization, conductance, data interpretation and analysis, and writing first draft. RK, SV, and MP: study conductance, data interpretation and analysis, and revising the manuscript.

### Conflict of Interest

The authors declare that the research was conducted in the absence of any commercial or financial relationships that could be construed as a potential conflict of interest.

## Abbreviations

EEG: electro-encephalography
mRS: modified Rankin Scale
NIHSS: National Institutes of Health Stroke Scale
REM: rapid eye movement.
